# Role of Faculty Development Programs in Medical Education at the University of Zimbabwe College of Health Sciences, Zimbabwe

**DOI:** 10.29024/aogh.5

**Published:** 2018-04-30

**Authors:** Antony Matsika, Kusum Nathoo, Margaret Borok, Thokozile Mashaah, Felix Madya, Susan Connors, Thomas Campbell, James G. Hakim

**Affiliations:** 1 University of Zimbabwe College of Health Sciences, Avondale, Harare, ZW

## Abstract

**Background:**

Major challenges are being experienced in medical education in sub-Saharan African Universities. These include emigration of faculty, infrequent curriculum review, inadequate training in medical education, poor investments in infrastructure and lack of faculty development programs. The USA government committed funding to improve the quality of medical education and research capacity in sub-Saharan Africa through the Medical Education Partnership Initiative (MEPI).

**Objectives:**

This article describes the implementation of faculty development at the University of Zimbabwe College of Health Sciences (UZCHS), a recipient of a MEPI award.

**Methods:**

Data sources included annual surveys and reports of UZCHS MEPI activities, exit evaluation reports of faculty development workshops; results of a survey conducted in 2015 at the end of the MEPI grant. Questionnaires were developed based on the MEPI Zimbabwe evaluation plan and logic model. Surveys were administered to faculty members, postgraduate and undergraduate students. Qualitative data was collected through in-depth key informer interviews of stakeholder.

**Findings:**

Different faculty development activities were implemented such as workshops, exchange visits, visiting professors program, advanced leadership training and curriculum development. The implementation of the activities brought positive developments to the college as confirmed by faculty and students. The majority of faculty interviewed (96%) confirmed that faculty development programs were very helpful in enhancing their expertise and skills. A similar number, i.e. 96%, also reported satisfaction with the training.

**Conclusions:**

We have described how the implementation of faculty development programs at the UZCHS contributed to the improvement of medical education at the College. The short term and long-term benefits of faculty development have been analyzed. Various forms of faculty development programs were described. Limitations of this analysis were the inability to collect data on students’ performance and the demonstration of changes in teaching performance.

## Background

Faculty development enables faculty members to acquire skills that enhance their performance in their roles as teachers and researchers to improve student outcomes [1]. In most medical schools faculty members are employed as educators with limited professional training in education [2]. Rapid evolution in medical education methods and approaches requires faculty who are capable of embracing these changes [3,4]. Students are also demanding a better quality of education [5]. As a result there is greater recognition of the importance of the implementation of faculty development programs [6,7]. In addition, its justification emanates from its ability to improve teaching performance and better learning outcomes for students [7]. In light of this, most universities have invested heavily in enhancing faculty teaching competences [8,9]. Despite abundant evidence of the positive role of faculty development in medical education, at the UZCHS, there were only a few faculty development sessions taking place prior to the advent of the Medical Education Partnership Initiative (MEPI). The goal of this study is to describe how faculty development programs through MEPI impacted faculty and students, and contributed to addressing medical education challenges at the UZCHS.

## MEPI Program at the UZCHS

The Medical Education Partnership Initiative (MEPI) was funded (2010–2015) by the USA Presidential Emergency Plan for AIDS Relief (PEPFAR) and the National Institutes of Health (NIH) with implementation through the Fogarty International Centre (FIC) and Health Resource and Services Administration (HRSA) [10]. Thirteen medical schools in sub-Saharan Africa countries were funded, including the University of Zimbabwe College of Health Sciences (UZCHS). The aim was to improve health outcomes through increasing medical schools’ output of better trained health professionals, who would be able to generate evidence to support local interventions. Retention of health professionals and sustainability of initiatives were key goals of the initiative. UZCHS implemented the program in partnership with the University of Cape Town, University of Colorado-Denver, Stanford University, Kings College London and University College London. Under MEPI, UZCHS was awarded a programmatic award, Novel Education Clinical Trainees and Researchers (NECTAR), and cardiovascular disease and mental health linked awards.

A combination of political and economic challenges in Zimbabwe led to the stagnation of medical education development in the decade before the implementation of the MEPI program in 2010 [10,11]. Through the MEPI grant the university sought to redress the situation. In this article, we describe faculty development during the implementation of the MEPI program.

## Methods

The Evaluation Centre of University of Colorado-Denver in collaboration with UZCHS evaluators conducted a survey in 2015 for the MEPI program at the UZCHS [12]. Questionnaires were send out to all participants (i.e. faculty, postgraduate and undergraduate students who had benefited in one way or the other from faculty development programs). Forty-three faculty members (26%) who participated in faculty development, 39 postgraduate students (23%) and 408 undergraduate students (60%) responded. In addition, 37 out of 41 faculty who completed a one-year advanced faculty development certificate were surveyed. All 37 belonged to the UZCHS and the other 4 to other institutions. A pilot survey was conducted prior to the full administering of the questionnaires. The Lickert scale was used to capture respondents’ perceptions about faculty development. Questions were evaluated against the logic model to ensure that they were capturing the desired outcomes. Qualitative data was collected using key stakeholder in-depth interviews with 37 stakeholders comprising of MEPI leaders at UZCHS, Mentored Clinical Scholars Program attendees and 18 participants (faculty and masters students) who were nominated by program leadership as those who had received great benefit by participating in the activities. The 18 participants were interviewed in the profiles of progress so as to measure the impact of the programs. In addition, the evaluators also conducted observations during implementation of programs and reviewed relevant documents. SPSS was used for summarizing quantitative survey data for faculty development and HEALZ. Nvivo software was used to code and analyze interview and open response survey data.

Permission was granted from the Dean’s office to carry out the study. Informed consent was sought and obtained from all participants. Names of participants and responses were treated with confidentiality.

## Findings

A table was developed using data from the survey report, technical progress reports, visiting professors’ reports and faculty development workshops reports to summarize the implementation of faculty development programs (see Table 1 overleaf).

**Table 1:** Summary of Faculty Development Activities.

Strategy	Methodology	Competences	Facilitators	Outputs

Faculty Development	3 workshops per year Establishment of Health Professions Education department	Teaching methods Learner Assessments including MCQs Setting objectives and learning outcomes Team based learning Patient safety	Visiting Professors from University of Colorado-Denver (UCD) in collaboration with UZCHS faculty	13 Workshops between 2011–2015 69% (122 out of 166) attended 1 or more workshops 14 local faculty mentored in facilitation
	Mental Health-linked award master classes	Psychotherapy Child Psychiatry Cognitive Behavioral therapy Self-harm (team based learning) Griffiths Child Development Scales training Forensic Psychiatry HIV Adherence Community-based Psychiatry	University of Cape Town (UCT) External Consultants Local faculty Government officials	Increase from 1 (2010) to 7 lecturers in 2015 Introduction of mental health outreach services Establishment of special clinics such as Child Psychiatry clinics
		Faculty Exchange visits	UCT Stanford University	All faculty in the Psychiatry department visited South Africa & UK institutions
	Academic Leadership training through Southern Africa FAIMER Institute (SAFRI) and Health Education Advanced Leadership in Zimbabwe (HEALZ)	Adult education Change management Leadership Mentoring Curriculum development Program evaluation	UCD Visiting faculty for HEALZ Southern Africa FAIMER Institute (SAFRI)	4 Faculty members including the Dean of UZCHS completed the FAIMER course 41 participants completed HEALZ
Curriculum Development and implementation	Training of faculty through HEALZ Presentations by external experts on curriculum development	Development of new curricula Curriculum review Curriculum evaluation Implementation	Dean’s Office Visiting Faculty	New modules developed for Psychiatry undergraduates: depression, mood disorders, substance abuse Post graduate Psychiatry introduced subspecialty (Child forensic, community HVI/AIDS, Malaria and TB curricula established

## Core Faculty Development Workshops

Faculty development was implemented in the form of workshops focusing on relevant topics and other emerging issues (see Table 1, competences column). These were delivered by experts from University of Colorado-Denver in collaboration with local faculty who were mentored earlier in the program. Content of the workshops included curriculum development, learner assessments, principles of adult education, setting out objectives, team-based learning, and many others. Masterclass workshops were implemented for the mental health linked award and covered topics such as psychotherapy, community based psychiatry, forensic psychiatry, substance abuse, among others. Health Professions Education department was established in 2012 to sustain faculty development initiatives and also ultimately expand its horizons to offer diplomas and higher degrees.

Thirteen faculty development workshops were held between 2012 and 2015. Ten masterclasses were also conducted in the same period by the mental health award. Annual attendance is shown in the graph above. Attendance was at its peak in the second year and the fourth year was the least attended. Sixty-nine percent (122 out of 166) attended one or more of the 13 faculty development workshops. Faculty were awarded Continuous Professional Development (CPD) points and certificates for attending faculty development workshops offered from the second year. It was observed that faculty who are clinicians reported lower attendance records.

Figure 2 below shows five different perceptions by faculty regarding faculty development training: gaining new knowledge, improvement in teaching skills, intention to incorporate new methods, training satisfaction, and usefulness.

In 2015, 96% of faculty (43) who attended the workshops reported they had gained new knowledge from their participation. Ninety-one percent reported gaining new teaching skills. All of the faculty interviewed indicated they planned to incorporate new methods acquired in their instructional practice. On the usefulness of topics, 96% of faculty concurred that the topics will help to improve the quality of education offered at the College. Nineteen out of the 43 faculty also indicated that they had incorporated one or more of the methods or strategies presented during faculty development workshops in their instructional practice e.g. team based learning (n = 11) and student assessment methods (n = 8).

**Figure 1:**
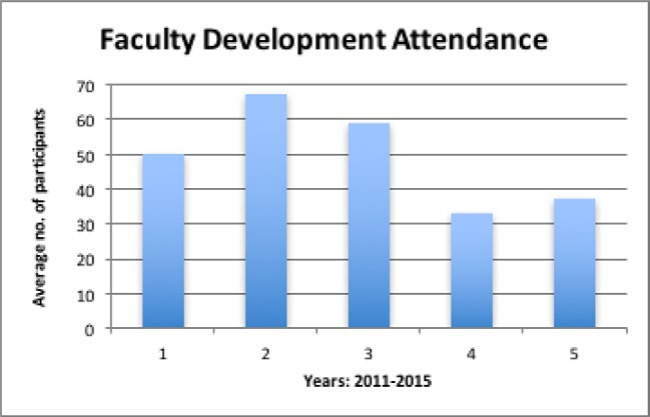
Yearly Average Attendance of Faculty Development Workshops.

**Figure 2:**
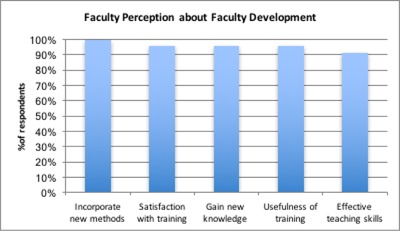
Benefits of Faculty Development.

One of the beneficiaries of faculty development workshops had this to say:

‘I attended the team-based learning workshop. My life was never the same in terms of teaching … I made an overhaul to one of my courses to make it team-based learning. I cover it in an amazingly short period of time, and the students have benefited. Students’ evaluation of the course at the end of the course was amazing this time around.’

In confirming the impact of faculty development one interviewee said:

‘We had been teaching mainly the way we were taught and most of us had no idea really what was happening there. Now with the faculty development programs, we now appreciate… what the curriculum is all about, how to teach and how to assist our students.’

## Advanced Leadership Training

The Health Education Advanced Leadership in Zimbabwe (HEALZ) program was established to continuously develop academic leadership capable of leading positive change and health education reforms. This is a year-long certificate program implemented by the department of Health Professions Education, consisting of training and capacity building in academic leadership, curriculum development and review, and program evaluation. The objective is to create future leaders who are capable of carrying out curricula reforms, independent research and program evaluations in education programs. Forty-one participants completed the HEALZ program in three cohorts of 13 to 15 faculty members from 2013 to 2015. All 37 participants from the UZCHS, when interviewed, reported they were ‘satisfied’ or ‘extremely satisfied’ with the professional development they went through in the HEALZ program. They also reported gaining confidence and competence in leadership and interpersonal skills e.g. enhanced communication and improved interactions.

One HEALZ scholar said, “At a personal level, I have become more confident in what I do and how I communicate because of the HEALZ training.”

Another HEALZ beneficiary confirming the immediate benefits of the training said:

“I think I am now a better teacher. You have clear learning objectives. You lay out what you want the students to know, and this is how you can examine them to get that information. … Also we went through how they learn, different approaches. … This is my second year applying some of the techniques. …. The students enjoyed the lectures. … They were contributing.”

Four UZCHS faculty members, including the dean, completed a two-year fellowship program in medical education offered by Southern Africa FAIMER Institute. Content of the program included advanced academic leadership, curriculum development and evaluation, change management and mentoring.

## Faculty Practice

### Evidence of implementation of new methods learnt

Faculty were also requested to make self-assessments on their faculty practice, focusing on the quality of their educator skills and student assessment strategies. In 2015, 81% reported advanced or expert high-quality teaching practices and similarly 83% reported advanced or expert skills in conducting students’ assessments using new methods.

Undergraduate students were interviewed in order to gather evidence of implementation of content of faculty development by faculty.

In 2015, 21% of the students interviewed indicated that they received feedback on their performance from faculty besides examination grades. 75% reported that they were provided with course objectives at the beginning of the course. Only 37% thought that they had enough opportunity to practice what they had learnt.

**Figure 3:**
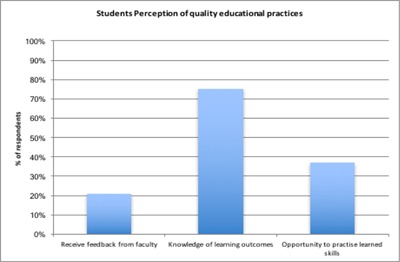
Students’ Perception About the Quality of Practices.

**Figure 4:**
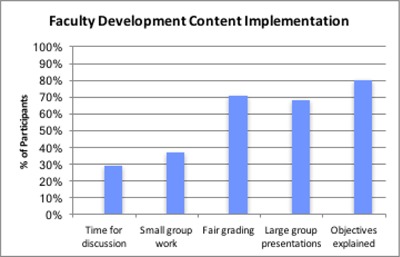
Students’ Responses on the Implementation of Faculty Development Content by Faculty.

In 2015, students were interviewed on whether five selected faculty development contents were being implemented. Eighty percent of the students acknowledged lecturers were explaining objectives of lectures prior to the sessions. On the other hand, only 29% and 37% acknowledged time for discussion and small group work respectively were being implemented.

## Curriculum Development

Seventy-nine percent of faculty interviewed on curriculum development indicated that they had begun to review their curriculum, whilst 74% reported advanced or expert skills in curriculum development. However only 12% reported they had concrete plans to revise their curriculum.

## Impact of the Program on Individuals

The impact of the program was measured on a nine-point criteria as outlined in the table below using 18 faculty respondents’ responses ranked in order of frequency.

Participants indicated that there had been enhanced quality of medical education due to faculty development workshops. One interviewee reported on being a ‘better teacher’:

“The program introduced us to different methods of teaching and team-based learning so that students … can also participate … It has enhanced my experiences in teaching, and it has made it more interesting… The students present and we discuss… and we end up with something very comprehensive. The student participation has been advanced a lot.”

In confirming the impact on individuals yet another interviewee had this to say:

“It has helped my students [be] more interested in what we are learning. It has also made me enjoy what I’m doing. We also have discussions on examinations, like on setting up MCQs… It helped me to be a better examiner to my students and it helped me to be able to help me to enjoy my job as faculty also.”

**Figure 5:**
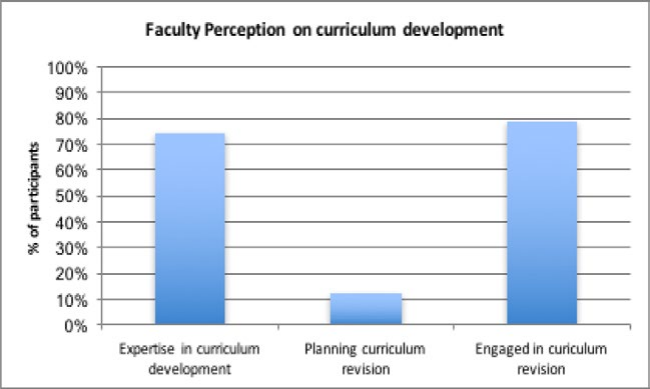
Faculty Perception on Curriculum Development.

## Discussion

The faculty development programs were implemented in collaboration with regional and international academic institutions [12]. This is supported by some authors like Steynert in 2012 who commented on the fostering of new partnerships and collaborations to effectively implement programs [6]. The faculty development programs implemented by MEPI transformed the landscape of medical education at the college. Faculty development programs were institutionalized through the establishment of the Department of Health Professions Education to support sustainability of the programs. New curricula for HIV/AIDS, malaria and tuberculosis were developed. Similarly, new curricula and modules were developed for psychiatry undergraduates and postgraduates. New services were introduced in the Psychiatry department such as mental health outreach and the Child Psychiatry clinic. The dean, leading by example together with three other senior lecturers, completed a fellowship program in health professions education specializing in advanced leadership offered by the Southern Africa FAIMER Institute. Fourteen local faculty were mentored in facilitation by visiting faculty from UCD and Stanford University. These mentored faculty have taken over the facilitation of faculty development programs. The MEPI-Zimbabwe program implemented a number of faculty development programs that included workshops, mentorship, exchange visits, seminars/guest lectures and training courses [12]. This is consistent with some studies that have listed some of these programs as part of faculty development [2].

The findings consistently show high satisfaction of the majority of faculty members with faculty professional development activities. Change in attitudes and perceptions, gaining new knowledge and skills, increased confidence, implementing new instructional practices, expanded professional networks and infrastructural investments were some of the positive impacts reported. These findings conform to other research that has concluded that in general faculty feel positive about the effectiveness of faculty professional development and that they yield positive outcomes in teacher practices and student learning [6,7]. Table 2 of the findings illustrates the extent faculty development programs have positively impacted on medical education.

**Table 2:** 

No.	Impact	No. of responses. N = 18	Comment

1	Better teacher	15	*Indicated improved teaching abilities e.g. increase use of interactive teaching methods*.
2	Increased confidence	12	*Indicated increase in confidence across various roles as teachers, clinicians and researchers*.
3	Expanded professional networks	12	*Reported appreciation of the expanded networks created with international and local professionals. Developed a new sense of connection*.
4	Increased interest in teaching	8	*Increased interest in teaching as a career*.
5	Increased interest in research	8	*Indicated development of new interest in a research career*.
6	Better clinicians	8	*Indicated improved effectiveness as clinicians especially with the introduction of new technology and e-resource*.
7	New career pathways	8	*Reported program supported them in development of a clear career path*.
8	Better researcher	7	*Reported enhancement of research skills*.
9	Improved interpersonal interactions	4	*Indicated positive influence on their interpersonal interactions both professionally and personally*.

The MEPI program adopted a four-cluster model (Kala, 2015) i.e. professional (individual expertise), instructional (teaching skills), curricular (curriculum design and delivery) and organizational development (institutional practices) to implement faculty development programs [7]. In addition to the commonly mentioned faculty development programs, the MEPI program expanded to include exchange visits of faculty with partner academic institutions to share best practices [13]. A visiting professors’ program was implemented where faculty from partner institutions traveled to University of Zimbabwe to facilitate faculty development workshops, teach students, conduct guest lectures and mentor local faculty [12]. The training of faculty in academic leadership through the HEALZ program was necessary to enhance faculty capabilities to influence change in the institution through skills like curriculum planning, stimulating and managing curricular change and establish critical mass of academic leaders [2].

Despite the positive attributes of faculty development programs, attendance by faculty was inconsistent (Figure 1). This may have resulted because in the first year the program was new and faculty may have been unsure about the benefits of the workshops. In the second year, a combination of intensive advocacy, compulsory attendance and effective change management strategies helped to increase the attendance numbers. In years four and five there was a drastic drop in attendance signifying perhaps the end of MEPI. However, we did not conduct studies to determine the reasons for this trend.

Again, this study did not include assessment of the performance of students to determine outcomes, hence creating the need for further studies to establish the relationship between students performance and faculty development. Similarly, the evaluation exercise did not include the assessment of performance of faculty in classroom except for feedback from students on implementation of content and new learned practices. This is despite studies by Steynert in 2006 and Kala in 2015 that showed that faculty development improves teaching performance and better learning outcomes for students. The attributes for this include; development of new teaching skills, new assessment techniques, better ways of planning or implementing curricular, new ways of student-teacher relationships and increased commitment to educational scholarships.

## Conclusions

We have demonstrated the role of faculty development programs through their short-term impacts on teaching skills, knowledge acquisition, change in attitudes, leadership practices, workshops attendance and perceptions in general. Long term benefits resulting from faculty development have been described as increased confidence, expanded professional networks, interest in teaching, interest in research and better clinicians among others.

We recommend some strategies to improve faculty development programs. First, faculty development programs should be streamlined according to disciplines/departments e.g. topics and courses to achieve greater participation. However, this militates against inter-professional learning. Second, to ensure effectiveness of faculty development programs in resource limited settings, resources may be prioritized to areas of greatest impact in faculty development. Third, all faculty members should be mandated to attend faculty development programs. Fourth, where external funding is the main source of support for faculty development programs, a sustainability plan is necessary to maintain the momentum after the end of funding.
